# Association of physical activity, sedentary behaviour, and daylight exposure with sleep in an ageing population: findings from the Whitehall accelerometer sub-study

**DOI:** 10.1186/s12966-022-01391-0

**Published:** 2022-12-09

**Authors:** Quentin Le Cornu, Mathilde Chen, Vincent van Hees, Damien Léger, Aurore Fayosse, Manasa S. Yerramalla, Séverine Sabia

**Affiliations:** 1 Inserm U1153, CRESS, Epidemiology of Ageing and Neurodegenerative diseases, Université de Paris Cité, 10 avenue de Verdun, 75010 Paris, France; 2 Accelting, Almere, The Netherlands; 3 Department of Epidemiology and Public Health, University College London, London, UK; 4 APHP, Hôtel-Dieu, Consultation de pathologie professionnelle Sommeil Vigilance et Travail, Centre du Sommeil et de la Vigilance, Paris, France

**Keywords:** Sleep, Physical activity, Sedentary time, Daylight exposure, Older adults, Accelerometer

## Abstract

**Background:**

Ageing is accompanied by changes in sleep, while poor sleep is suggested as a risk factor for several health outcomes. Non-pharmacological approaches have been proposed to improve sleep in elderly; their impact remains to be investigated. The aim of this study was to examine the independent day-to-day associations of physical behaviours and daylight exposure with sleep characteristics among older adults.

**Methods:**

Data were drawn from 3942 participants (age range: 60–83 years; 27% women) from the Whitehall II accelerometer sub-study. Day-to-day associations of objectively-assessed daytime physical behaviours (sedentary behaviour, light-intensity physical activity (LIPA), moderate-to-vigorous physical activity (MVPA), mean acceleration, physical activity chronotype) and daylight exposure (proportion of waking window with light exposure > 1000 lx and light chronotype) with sleep characteristics were examined using mixed models.

**Results:**

A 10%-increase in proportion of the waking period spent sedentary was associated with 5.12-minute (4.31, 5.92) later sleep onset and 1.76-minute shorter sleep duration (95%confidence interval: 0.86, 2.66). Similar increases in LIPA and MVPA were associated with 6.69 (5.67, 7.71) and 4.15 (2.49, 5.81) earlier sleep onset respectively and around 2-minute longer sleep duration (2.02 (0.87, 3.17) and 2.23 (0.36, 4.11), respectively), although the association was attenuated for MVPA after adjustment for daylight exposure (1.11 (− 0.84, 3.06)). A 3-hour later physical activity chronotype was associated with a 4.79-minute later sleep onset (4.15, 5.43) and 2.73-minute shorter sleep duration (1.99, 3.47). A 10%-increase in proportion of waking period exposed to light> 1000 lx was associated with 1.36-minute longer sleep (0.69, 2.03), independently from mean acceleration. Associations found for sleep duration were also evident for duration of the sleep windows with slightly larger effect size (for example, 3.60 (2.37, 4.82) minutes for 10%-increase in LIPA), resulting in associations with sleep efficiency in the opposite direction (for example, − 0.29% (− 0.42, − 0.16) for 10%-increase in LIPA). Overall, associations were stronger for women than for men.

**Conclusions:**

In this study, higher levels of physical activity and daylight exposure were associated with slightly longer sleep in older adults. Given the small effect sizes of the associations, increased physical activity and daylight exposure might not be enough to improve sleep.

**Supplementary Information:**

The online version contains supplementary material available at 10.1186/s12966-022-01391-0.

## Introduction

Around one third of human life is spent sleeping, emphasizing the importance of sleep for health [1–6]. As people get older, they tend to sleep less and in a more fragmented manner [7–9]. Current recommendations for sleep relate to sleep duration and sleep hygiene [10]. At older ages it is recommended to sleep 7 to 8 hours per night, to maintain regular sleep timing, and reduce light exposure at bedtime [10, 11]. Pharmacological treatments to improve sleep are available, but their usage is debated due to adverse effects such as excessive daytime sleepiness or poor motor coordination, both of which may lead to injury and falls at night [12, 13]. Non-pharmacological approaches to improve sleep, such as physical activity and light exposure, are thus increasingly being considered [14, 15], including among older adults [16].

A review of interventional studies among older adults reported that 12-week to 6-month moderate intensity exercise programs were associated with improved sleep quantity and quality [16]. However, when examining findings from the three studies that used objective sleep measures (polysomnography or accelerometer), there was no clear evidence of improved sleep following the interventions. Few observational studies have examined the association between physical activity and sleep characteristics using objective measures in older adults. Two of them found a day-to-day association of physical activity with longer sleep duration but not better sleep quality as measured by sleep efficiency or fragmentation [17, 18]. Some other studies, but not all [19], found weekly average physical activity to be associated with better sleep quality [20–23], with stronger evidence for light-intensity than moderate physical activity [20, 21]. Several studies have also reported that longer sedentary time might be deleterious for sleep duration [24] and quality [19, 25] although the strength of the association varies between studies. Given the potential importance of physical behaviours (sedentary behaviour (SB), light-intensity physical activity (LIPA), and moderate-to-vigorous physical activity (MVPA)) for sleep, it is important to consider the full spectrum of activity intensities [19, 21, 24].

Light is thought to be a core environmental cue of sleep-wake cycle [14, 26]. Increased daylight exposure, particularly in the morning, is suggested to be associated with longer sleep duration and better sleep quality [14]. Most of the evidence in this domain comes from experimental studies that show bright light (> 1000 lx) to be more strongly associated with sleep outcomes [14]. However, whether this association is consistent in day life setting and across the lifespan deserves further investigation [14].

Overall, the role of physical behaviours and daylight exposure for sleep, particularly among older adults, remains unclear for several reasons. First, there is heterogeneity in measurement tools that have been used to assess light exposure [14], physical behaviours [16], and sleep [15], some being based on self-reports leading to potential measurement bias [16]. Second, although a bidirectional association between physical behaviours and sleep is suggested [17, 18], measures of physical behaviours, daylight exposure, and sleep have often been averaged over several days, ignoring the day-to-day intra-individual variability and the temporality of the association [19–24, 27]. Third, physical behaviours and daylight exposure have never been examined together in relation to sleep, precluding conclusions to be drawn regarding their independent role. Fourth, physical behaviours [28] and sleep [29] patterns differ by sex and daylight exposure depends on season, highlighting the importance to examine whether the association between physical behaviours, daylight exposure, and sleep is modified by sex or season. Finally, most studies on older adults were based on small sample size [17–19, 21, 24] limiting generalisation of findings in this age group.

This study aims to examine the day-to-day association of physical behaviours and daylight exposure with sleep characteristics using data from 3942 older adults of the Whitehall II accelerometer sub-study. In order to assess robustness of findings, we also investigated the independence of associations of physical behaviours and daylight with sleep, and whether associations were similar in men and women or by season of wear.

## Methods

### Study population

The Whitehall II prospective cohort study was established in 1985–1988 among 10,308 British civil servants (33% women) aged 35–55 years at enrolment [30]. Since inception, sociodemographic, behavioural and health-related factors have been assessed using questionnaires and clinical examinations approximately every four-five years. An accelerometer measure was added to the 2012–2013 wave of data collection for participants (age range: 60 to 83 years) seen at the London clinic and those living in the South-Eastern regions of England who underwent clinical examination at their home, constituting the population of the present study. At each wave, participants provided written informed consent and research ethics approvals were obtained from the University College London ethics committee (latest reference number 85/0938).

### Physical behaviours, daylight, and sleep measurements

At the 2012–2013 clinical examination, participants were asked to wear a triaxial accelerometer (GENEActiv Original; Activinsights Ltd., Kimbolton, UK) on their non-dominant wrist for 9 consecutive, 24-hour, days. Over the period of the accelerometer wear, they also completed a daily sleep log answering the following questions: “What time did you first fall asleep last night?” and “What time did you wake up today (eyes open, ready to get up)?”. The device also included a light sensor that captures light in the visible range of wavelength (silicon photodiode sensor, 400–1100 nm wavelength range, 0–3000 lx range, 5-lx resolution) [31].

Accelerometer data sampled at 85.7 Hz, with acceleration expressed relative to gravity (1 *g* = 9.81 m/s^2^), were processed using GGIR R-package [32] (version 2.4–0). Euclidean Norm of raw accelerations Minus One, with negative values rounded to zero, were calculated [33] and averaged over 60-second epochs. Sleep episodes were identified using a validated algorithm guided by the sleep log [34]. Data from the first waking up (day 2) to waking up on the day before the last day (day 8) were used. This resulted in 7 full days (waking-to-waking windows) of data per participant, corresponding to 7 waking (from wake up to start the day to sleep onset at night) and sleep (from sleep onset at night to the following wake up to start the day) windows. Participants were included for analyses if both wear times during waking window and the following sleep window corresponded to ≥2/3 of the respective windows [35]. Non-wear period among valid days was corrected based on a previously reported algorithm [33].

#### Physical behaviours during waking window

Five physical behaviours variables were extracted for each waking window. *Mean acceleration* (in m*g*) was used as a marker of global activity level. *Proportions of waking window spent in SB, LIPA, and MVPA* were calculated as the time accumulated in average acceleration over a 60-s epoch < 0.04 *g*, ≥0.04 and < 0.1 *g*, and ≥ 0.1 *g*, respectively [36, 37], over the waking window divided by duration of the waking window. *Timing of the five most active hours* was used to represent physical activity chronotype. In analyses, we examined the association for a 10%-increase in the proportions of waking window in SB, LIPA, and MVPA, for 10 m*g* increase in mean acceleration and 3-hour increase in physical activity chronotype.

#### Daylight exposure during waking window

We used the intensity threshold of 1000 lx to differentiate indoor and outdoor light as in previous studies [14, 38]. For each waking window, two markers of daylight exposure were extracted: the *proportion of waking window with light exposure > 1000 lx* calculated as the accumulated time in 15-min epochs with peak value > 1000 lx over the waking window divided by the duration of waking window; the *chronotype of daylight exposure* corresponding to the period of the day when the person is most exposed to outdoor light, estimated as the 4-hour window (among 8-12 h, 12-16 h, and 16-20 h windows) with highest duration in light exposure > 1000 lx and in case of equal duration between two windows, the one with highest mean light exposure was selected. In analyses, we examined the association for a 10% increase in the proportion of waking window with light exposure > 1000 lx and for the 3 categories of light chronotype: Morning (8-12 h, reference), Afternoon (12-16 h), and Evening (16-20 h).

#### Sleep

For each sleep window, the following sleep characteristics were considered: *sleep onset* (time when the person fell asleep to start the night, in minutes), *duration of sleep window* (time difference between sleep onset and next waking to start the day, in minutes), *sleep duration* (time slept during the sleep window, as defined by no change in arm angle greater than 5° for 5 minutes or more, in minutes), and *sleep efficiency* (here sleep duration divided by duration of the sleep window, in percent) [34].

### Covariates

Covariates were drawn from questionnaire and clinical examination during the 2012–2013 wave of data collection as well as from electronic health records (Hospital Episode Statistics (HES), cancer registry, and the Mental Health Services Data Set). *Sociodemographic variables* comprised age, sex, ethnicity (white, non-white), marital status (married/cohabiting, other), level of education (≤primary school, lower secondary school, higher secondary school, university, or higher degree; treated as ordinal variable), and professional activity status (active, inactive people). *Wearing time periods* included day type (week days, weekend) and season of wear (autumn/winter (from September equinox to March equinox) or spring/summer (from March equinox to September equinox)). *Behavioural variables* were smoking status (never smoker, ex-smoker, current smoker), alcohol consumption (none, 1–14 units/week, > 14 units/week), fruits and vegetables consumption (<daily, daily, >daily), and nap habits (no, yes). *Health-related variables* included body mass index (BMI; categorized as < 25, 25–29.9, and ≥ 30 kg/m^2^), self-reported medications known to impact sleep (corticosteroids, hypnotics, anxiolytics, antidepressants, antipsychotics), as referred thereafter as sleep medication for ease of reading, and number of chronic conditions among diabetes (fasting glucose ≥7.0 mmol/L, self-reported doctor-diagnosed diabetes, use of anti-diabetic medications, or record in HES), coronary heart disease, stroke, heart failure, cancer, arthritis, chronic obstructive pulmonary disease, depression, dementia, and Parkinson’s disease (assessed using HES records and data collected at Whitehall clinical exams as well as mental health records for depression and dementia).

### Statistical analyses

For descriptive analysis, we averaged physical behaviours, daylight, and sleep variables over the days of the week to obtain weekly averaged daily estimates for each participant. We showed characteristics of the population according to median groups of average daily mean acceleration, average daily proportion of waking window with light exposure > 1000 lx, and average daily sleep duration. We also reported averaged physical behaviours and light variables by median groups of sleep characteristics.

Then, we used linear mixed models to assess day-to-day association of physical behaviours and daylight exposure with sleep characteristics. This method is suited for nested data with repeated measures among the same individuals, the random effects account for the within-person variability over the days (waking-to-waking windows) of the observational period. For each individual and each of its waking-to-waking windows (composed of the waking window and sleep window), the exposure is the physical behaviours/daylight variables during the waking window and the outcome the sleep variable during the sleep window, so that the exposure precedes the outcome, accounting for the temporality of the association. We examined the association between tertiles of physical behaviours and daylight exposure with sleep characteristics. In absence of evidence of non-linearity, we conducted further analyses using standardised values of physical behaviours and daylight exposure variables as continuous terms. We first examined the associations of physical behaviours and daylight variables with sleep outcomes in separate models, using three levels of adjustment. Model 1 was adjusted for sociodemographic factors, season of accelerometer wear, and day type, Model 2 was additionally adjusted for behavioural factors and Model 3 was also adjusted for health-related variables. For the association between daylight and sleep, we used an additional 4th model mutually adjusted for daylight exposure and chronotype. Finally, we examined the independence of associations of physical behaviours and daylight exposure with sleep by adjusting Model 3 for light exposure or mean acceleration, respectively.

#### Sensitivity analyses

We conducted three sensitivity analyses. One, we tested interactions with sex and season of accelerometer wear. When significant interactions were found, we repeated analyses separately in each group. Second, we repeated the analysis with mutual adjustment of physical behaviours and daylight exposure including also daylight chronotype. Three, we excluded participants using sleep medication and those with depression, as both these aspects are likely to strongly impact physical behaviours [39], daylight exposure [40], and sleep [41]. Four, we restricted analyses to individuals with sleep problems but not using sleep medication, based on two definitions of sleep problems in absence of clinical diagnosis: Jenkins sleep problem score ≥ 12 [42] and accelerometer-derived sleep efficiency< 80% [43]. All analyses were undertaken using R software version 4.0.5 (http://www.r-project.org). Linear mixed-models were fitted using the function lme() from the lmerTest package version 3.1–3 [44]. A two-sided *P* < 0.05 was considered statistically significant.

## Results

### Population characteristics

**Fig. 1:**
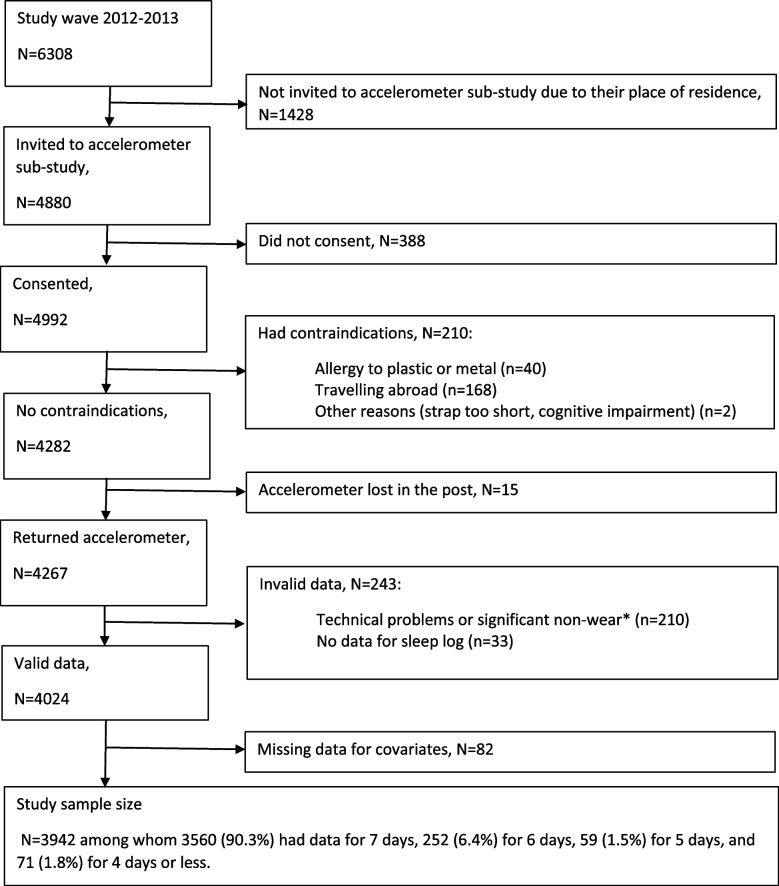
Flow chart of study sample selection. *Significant non-wear corresponds to ≥2/3 of wear time over the waking window and over the sleep window

Of the 4880 participants invited to participate in the accelerometer sub-study, 4492 consented to participate and 4024 had valid accelerometer data. Of them, 82 were excluded due to missing data for covariates. Our analysis was therefore based on 3942 participants, corresponding to 26,943 observational waking-sleep periods (flowchart in Fig. 1). Among participants included in the analysis, 90.3% (*N* = 3560) had valid accelerometer data for 7 days, 6.4% (*N* = 252) for 6 days, and 3.3% (*N* = 127) for 5 days or less. Compared to participants invited to take part to the accelerometer sub-study but not included in the analyses (*N* = 938), included participants were more likely to be men (74.0% vs 67.5%, *p* < 0.001), white (92.7% vs 89.4%, *p* = 0.001), and from lower educational group (69.3% vs 62.5%; *p* < 0.01).

Among the 3942 participants included in the analysis, the medians (interquartile range) of daily acceleration, proportion of waking time with light exposure > 1000 lx, and sleep duration were 30.8 (25.3–37.3) m*g*, 9.7 (3.7–20.3) %, and 6h39min (6h00min-7h13min), respectively. Characteristics of the study population according to the median of these variables are presented in Table 1. When considering data averaged over the days of the observational period per participant, most sociodemographic, behavioural, and health-related variables were associated with at least two of mean acceleration, daylight exposure, and sleep duration. Supplementary Table 1 shows mean of weekly averaged person-level physical behaviours and daylight variables by median of sleep characteristics. There was no clear association between physical behaviours and sleep characteristics, apart for sleep timing. In contrast, participants with higher mean sleep duration were more likely to be exposed to light > 1000 lx. In absence of evidence of non-linearity, we conducted the analysis using physical behaviours and daylight exposure variables as continuous terms (Supplementary Table 2).

**Table 1:** Population characteristics by daily acceleration, daylight exposure and sleep duration (*N* = 3942)

Characteristics	Daily mean acceleration^**a**^			% waking window > 1000 lx^**a**^			Average sleep duration^**a**^		
	< 30.8 m*g*^b^	≥ 30.8 m*g*^b^	*p*	< 9.7%^b^	≥ 9.7%^b^	*p*	< 6h39min^b^	≥ 6h39min^b^	*p*
Age (years), M (SD)	70.9 (5.8)	67.8 (5.1)	< 0.001	69.6 (5.8)	69.0 (5.5)	0.001	69.4 (5.7)	69.2 (5.7)	0.314
Women	514 (26.1)	509 (25.8)	0.884	344 (17.5)	679 (34.4)	< 0.001	451 (22.9)	572 (29.0)	< 0.001
Non-white ethnicity	182 (9.2)	107 (5.4)	< 0.001	159 (8.1)	130 (6.6)	0.087	169 (8.6)	120 (6.1)	0.003
High school diploma or above	577 (29.3)	634 (32.2)	0.026	678 (34.4)	533 (27.0)	< 0.001	611 (31.0)	600 (30.4)	0.487
Currently employed	304 (15.4)	446 (22.6)	< 0.001	397 (20.1)	353 (17.9)	0.081	412 (20.9)	338 (17.1)	0.003
Married/cohabiting	1428 (72.5)	1516 (76.9)	< 0.001	1461 (74.1)	1483 (75.2)	0.442	1416 (71.9)	1528 (77.5)	< 0.001
Current smokers	86 (4.4)	42 (2.1)	< 0.001	69 (3.5)	59 (3.0)	0.651	71 (3.6)	57 (2.9)	0.418
1–14 alcohol units/week	1104 (56.0)	1131 (57.4)	< 0.001	1120 (56.8)	1115 (56.6)	0.384	1121 (56.9)	1114 (56.7)	0.949
Daily fruit and vegetable consumption	1504 (76.3)	1630 (82.7)	< 0.001	1542 (78.2)	1592 (80.8)	0.053	1532 (77.8)	1602 (81.2)	0.008
Having naps	1388 (70.4)	1185 (60.1)	< 0.001	1323 (67.1)	1250 (63.4)	0.016	1413 (71.7)	1160 (58.8)	< 0.001
Body mass index ≥30 kg/m^2^	488 (24.8)	222 (11.3)	< 0.001	353 (17.9)	357 (18.1)	0.267	399 (20.3)	311 (15.8)	< 0.001
Use of sleep medication	302 (15.3)	245 (12.4)	< 0.001	281 (14.3)	266 (13.5)	0.519	272 (13.8)	275 (13.9)	0.937
Number of chronic conditions^c^, M (SD)	0.8 (0.9)	0.5 (0.7)	< 0.001	0.7 (0.9)	0.6 (0.8)	0.007	0.7 (0.9)	0.7 (0.9)	0.557
Mean acceleration (m*g*), M (SD)	24.6 (4.4)	39.2 (7.9)	< 0.001	29.5 (8.8)	34.3 (10.0)	< 0.001	31.8 (9.7)	32.0 (9.8)	0.635
% waking window > 1000 lx, M (SD)	10.2 (9.8)	17.2 (13.9)	< 0.001	4.1 (2.8)	23.3 (11.0)	< 0.001	13.1 (12.1)	14.2 (12.9)	0.005
Sleep duration (minutes), M (SD)	394.6 (60.0)	393.1 (54.0)	0.429	393.0 (58.5)	394.8 (55.7)	0.324	349.8 (41.8)	437.9 (29.9)	< 0.001

Data are N (%), otherwise stated. *M* mean, *SD* standard deviation

^a^Data were averaged over the days of the observation period

^b^Median value in the study sample

^c^Chronic conditions include diabetes, coronary heart disease, stroke, heart failure, cancer, arthritis, chronic obstructive pulmonary disease, depression, dementia, and Parkinson’s disease

### Association between physical behaviours and sleep

Table 2 shows day-to-day associations between physical behaviours and sleep characteristics using mixed-effect models. In fully adjusted models, 10% increase in proportion of waking window spent in SB was associated with a 5.12 (95% confidence interval (95%CI): 4.31, 5.92) minutes later sleep onset. It was also associated with shorter sleep duration (− 1.76 [− 2.66, − 0.86] minutes) and duration of the sleep window (− 2.81 [− 3.77, − 1.85] minutes), resulting in a slightly better sleep efficiency (0.21 [0.11, 0.31] %)). For both proportions of the waking window in LIPA and in MVPA, an increase of 10% was associated with earlier sleep onset (− 6.69 [− 7.71, − 5.67] and − 4.15 [− 5.81, − 2.49] minutes, respectively), longer sleep duration (2.02 [0.87, 3.17] and 2.23 [0.36, 4.11] minutes) and sleep window (3.60 [2.37, 4.82] and 2.64 [0.63, 4.65] minutes). Similar but weaker associations were found for mean acceleration. Later timing of the five most active hours was associated with later sleep onset, shorter sleep duration, and sleep window (difference [95%CI] for a 3-hour increase in timing of the five most active hours: 4.79 [4.15, 5.43], − 2.73 [− 3.47, − 1.99], and − 3.19 [− 3.99, − 2.39], respectively).

**Table 2:** Day-to-day association of physical behaviours with sleep characteristics

	Sleep onset (min)		Sleep duration (min)		Duration of sleep window (min)		Sleep efficiency (%)	
	Beta (95% CI)^a^	*p*	Beta (95% CI)^a^	*p*	Beta (95% CI)^a^	*p*	Beta (95% CI)^a^	*p*
**Mean acceleration, per 10 m*g***								
Model 1	−3.43 (−4.14, − 2.73)	< 0.001	1.45 (0.66, 2.25)	< 0.001	2.05 (1.20, 2.90)	< 0.001	− 0.12 (− 0.21, − 0.03)	0.007
Model 2	− 3.33 (− 4.04, − 2.62)	< 0.001	1.30 (0.51, 2.10)	0.001	1.90 (1.05, 2.76)	< 0.001	− 0.13 (− 0.22, − 0.04)	0.004
Model 3	− 3.27 (− 3.99, − 2.56)	< 0.001	1.10 (0.30, 1.90)	0.007	1.91 (1.05, 2.76)	< 0.001	− 0.17 (− 0.26, − 0.08)	< 0.001
**% waking window in SB, per 10% increase**								
Model 1	5.28 (4.48, 6.08)	< 0.001	− 2.17 (− 3.07, − 1.28)	< 0.001	− 3.00 (− 3.96, − 2.05)	< 0.001	0.16 (0.06, 0.26)	0.002
Model 2	5.16 (4.36, 5.96)	< 0.001	− 1.98 (− 2.88, − 1.09)	< 0.001	− 2.79 (− 3.75, − 1.84)	< 0.001	0.17 (0.07, 0.27)	0.001
Model 3	5.12 (4.31, 5.92)	< 0.001	− 1.76 (− 2.66, − 0.86)	< 0.001	− 2.81 (− 3.77, − 1.85)	< 0.001	0.21 (0.11, 0.31)	< 0.001
**% waking window in LIPA, per 10% increase**								
Model 1	−6.86 (− 7.88, − 5.84)	< 0.001	2.45 (1.31, 3.60)	< 0.001	3.86 (2.64, 5.08)	< 0.001	− 0.25 (− 0.38, − 0.12)	< 0.001
Model 2	−6.74 (− 7.76, − 5.72)	< 0.001	2.24 (1.09, 3.38)	< 0.001	3.60 (2.38, 4.82)	< 0.001	− 0.25 (− 0.38, − 0.12)	< 0.001
Model 3	−6.69 (− 7.71, − 5.67)	< 0.001	2.02 (0.87, 3.17)	< 0.001	3.60 (2.37, 4.82)	< 0.001	− 0.29 (− 0.42, − 0.16)	< 0.001
**% waking window in MVPA, per 10% increase**								
Model 1	−4.49 (− 6.15, − 2.84)	< 0.001	2.91 (1.04, 4.78)	0.002	2.88 (0.88, 4.88)	0.005	− 0.04 (− 0.25, 0.17)	0.738
Model 2	−4.29 (− 5.94, − 2.64)	< 0.001	2.66 (0.80, 4.53)	0.005	2.64 (0.64, 4.64)	0.010	−0.05 (− 0.26, 0.16)	0.628
Model 3	−4.15 (−5.81, − 2.49)	< 0.001	2.23 (0.36, 4.11)	0.019	2.64 (0.63, 4.65)	0.010	−0.12 (− 0.33, 0.09)	0.249
**Timing of the most 5 active hours, per 3-hour increase**								
Model 1	4.77 (4.13, 5.41)	< 0.001	−2.72 (− 3.46, − 1.98)	< 0.001	−3.19 (− 3.99, − 2.39)	< 0.001	0.02 (− 0.06, 0.10)	0.610
Model 2	4.78 (4.14, 5.42)	< 0.001	− 2.72 (− 3.45, − 1.98)	< 0.001	−3.18 (− 3.99, − 2.38)	< 0.001	0.02 (− 0.06, 0.10)	0.612
Model 3	4.79 (4.15, 5.43)	< 0.001	− 2.73 (− 3.47, − 1.99)	< 0.001	− 3.19 (− 3.99, − 2.39)	< 0.001	0.02 (− 0.06, 0.10)	0.644

*SB* sedentary behaviour, *LIPA* light-intensity physical activity, *MVPA* moderate-to-vigorous physical activity, *CI* confidence interval

^a^Estimated using linear mixed-effects regressions

Model 1 adjusted for sociodemographic factors, season of wear, and day type (week-end vs week day)

Model 2 additionally adjusted for behavioural factors

Model 3 additionally adjusted for body mass index, use of sleep medication, number of chronic conditions

### Association between daylight and sleep

Table 3 shows the association of daylight exposure and chronotype with sleep. An increase of 10% in proportion of waking window spent at light exposure > 1000 lx was associated with earlier sleep onset (− 3.50 [− 4.06, − 2.95] minutes), longer sleep duration (1.49 [0.86, 2.12] minutes) and sleep window (2.10 [1.43, 2.78] minutes), and lower sleep efficiency (− 0.08 [− 0.15, − 0.01] %). Additional adjustment for daylight chronotype did not substantially change the association between daylight exposure and sleep variables (Model 4). People more exposed to light > 1000 lx in the afternoon were more likely to fall asleep later (1.90 [0.66, 3.15] minutes) than people more exposed to light > 1000 lx in the morning, association that remained evident after adjusting for daylight exposure over the entire waking window (2.76 [1.51, 4.01] minutes).

**Table 3:** Day-to-day association of daylight exposure and chronotype with sleep characteristics

	Sleep onset (min)		Sleep duration (min)		Duration of sleep window (min)		Sleep efficiency (%)	
	Beta (95% CI)^a^	*p*	Beta (95% CI)^a^	*p*	Beta (95% CI)^a^	*p*	Beta (95% CI)^a^	*p*
**% waking window with light exposure > 1000 lx, per 10% increase**								
Model 1	−3.50 (− 4.05, − 2.94)	< 0.001	1.59 (0.95, 2.22)	< 0.001	2.23 (1.55, 2.91)	< 0.001	−0.08 (− 0.15, 0.00)	0.036
Model 2	− 3.51 (− 4.06, − 2.96)	< 0.001	1.52 (0.89, 2.15)	< 0.001	2.10 (1.42, 2.78)	< 0.001	− 0.07 (− 0.14, 0.00)	0.046
Model 3	−3.50 (− 4.06, − 2.95)	< 0.001	1.49 (0.86, 2.12)	< 0.001	2.10 (1.43, 2.78)	< 0.001	−0.08 (− 0.15, − 0.01)	0.034
Model 4	− 3.64 (− 4.20, − 3.08)	< 0.001	1.49 (0.86, 2.13)	< 0.001	2.12 (1.43, 2.80)	< 0.001	− 0.08 (− 0.15, − 0.01)	0.032
**Daylight chronotype**^**b**^								
Model 1								
Morning	0.00 (ref)		0.00 (ref)		0.00 (ref)		0.00 (ref)	
Afternoon	1.88 (0.63, 3.12)	0.003	−0.11 (− 1.54, 1.32)	0.879	− 0.40 (− 1.96, 1.17)	0.619	0.02 (− 0.14, 0.18)	0.797
Evening	1.94 (0.08, 3.79)	0.041	−1.44 (−3.57, 0.69)	0.185	−2.15 (− 4.47, 0.18)	0.071	0.04 (−0.20, 0.27)	0.760
Model 2								
Morning	0.00 (ref)		0.00 (ref)		0.00 (ref)		0.00 (ref)	
Afternoon	1.89 (0.65, 3.14)	0.003	−0.16 (−1.59, 1.27)	0.828	−0.47 (−2.03, 1.10)	0.560	0.02 (−0.14, 0.18)	0.800
Evening	1.98 (0.12, 3.83)	0.036	−1.37 (−3.50, 0.76)	0.208	−2.00 (−4.33, 0.33)	0.092	0.03 (−0.20, 0.27)	0.787
Model 3								
Morning	0.00 (ref)		0.00 (ref)		0.00 (ref)		0.00 (ref)	
Afternoon	1.90 (0.66, 3.15)	0.003	−0.17 (−1.61, 1.26)	0.828	−0.48 (−2.04, 1.09)	0.551	0.02 (−0.14, 0.18)	0.816
Evening	1.99 (0.13, 3.84)	0.037	−1.39 (−3.53, 0.74)	0.208	−2.02 (−4.35, 0.31)	0.089	0.03 (−0.21, 0.27)	0.801
Model 4								
Morning	0.00 (ref)		0.00 (ref)		0.00 (ref)		0.00 (ref)	
Afternoon	2.76 (1.51, 4.01)	< 0.001	−0.53 (−1.97, 0.91)	0.470	−0.99 (−2.56, 0.58)	0.219	0.04 (−0.12, 0.20)	0.647
Evening	1.29 (−0.56, 3.14)	0.172	−1.10 (−3.24, 1.03)	0.312	−1.60 (− 3.93, 0.73)	0.179	0.02 (−0.22, 0.25)	0.900

^a^Estimated using linear mixed-effects regressions. Model 1 adjusted for sociodemographic factors, season of wear, and day type. Model 2 additionally adjusted for behavioural factors. Model 3 additionally adjusted for body mass index, use of sleep medication, and number of chronic conditions. Model 4 corresponds to Model 3 with mutual adjustment on % waking window with light exposure > 1000 lx and light chronotype

^b^Daylight chronotype corresponds to the 4-hour window with highest time > 1000 lx among the 8-12 h, 12-16 h, and 16-20 h windows. For 19 observations (0.07%) chronotype was not examined due to overlap. Among all observations (*N* = 26,943), 43.4% were classified as morning, 43.6% as afternoon, and 12.9% as evening light chronotype

### Independence of associations of physical behaviours and daylight with sleep

**Fig. 2:**
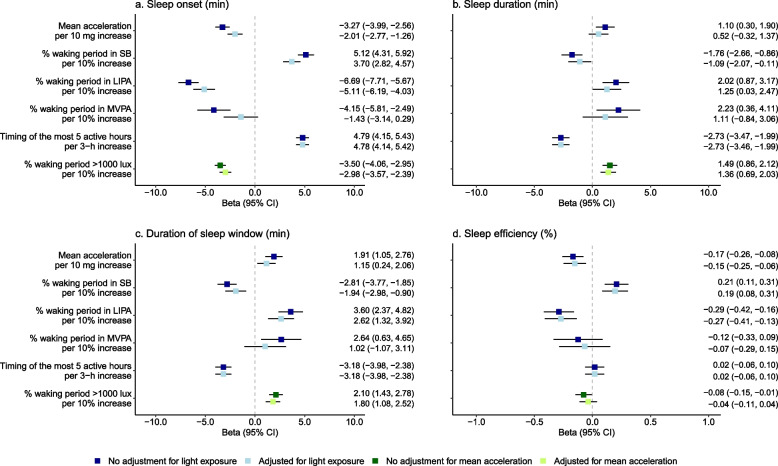
Independent day-to-day association of physical behaviours and daylight exposure with sleep characteristics. Models are adjusted for sociodemographic, behavioural, health-related factors. SB, sedentary behaviour; LIPA, light-intensity physical activity; MVPA, moderate-to-vigorous physical activity; CI, confidence interval

Adjustment for daylight exposure in models based on physical behaviours and for mean acceleration in models based on daylight exposure reduced the effect size of the associations observed for physical behaviours and daylight exposure respectively, and few lost significance (Fig. 2). Overall, after adjustment for daylight exposure > 1000 lx, associations of physical behaviours with sleep characteristics were reduced by 0.2 to 65.5% for sleep onset, 0.0 to 52.7% for sleep duration, 0.3 to 61.4% for duration of sleep window, and 6.9 to 11.8% for sleep efficiency. After adjustment for mean acceleration of the models for daylight exposure, the associations of daylight exposure > 1000 lx reduced by 14.9% for sleep onset, by 8.7% for sleep duration, by 14.3% for duration of sleep window, and became non-significant for sleep efficiency.

### Sensitivity analysis

Associations remained similar when models mutually adjusted for physical behaviours and daylight exposure were additionally adjusted for daylight chronotype (Supplementary Fig. 1) or when excluding participants using sleep medications and participants with depression (Supplementary Fig. 2). In analyses restricted to those with sleep problems, but not using sleep medication, associations were similar to those in the main analysis, although the association between physical behaviours and sleep characteristics were slightly stronger when using the sleep efficiency-based definition of sleep problems (Supplementary Figs. 3 and 4).

In order to allow comparison between the strength of associations of physical behaviours and daylight with sleep and the strength of associations with other covariates, Supplementary Table 3 shows the associations between covariates and sleep characteristics. The effect sizes of associations of physical behaviours and daylight exposure were small as compared to associations with other modifiable factors as reflected by the association of smoking with later sleep onset (25.95 [15.60, 36.30] minutes), and of having naps (− 17.38 [− 21.09, − 13.68] minutes), and obesity (− 14.09 [− 19.14, − 9.04] minutes) with sleep duration.

**Fig. 3:**
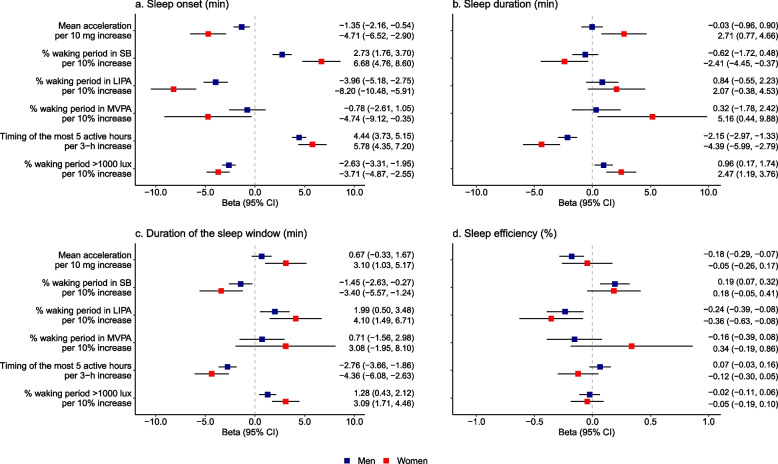
Day-to-day association of physical behaviours and daylight exposure with sleep characteristics separately in men and women. Models are adjusted for sociodemographic, behavioural, health-related factors, with additional adjustment for % waking window with light exposure > 1000 lux for models on activity behaviour variables, and for mean acceleration for model on light exposure. SB, sedentary behaviour; LIPA, light-intensity physical activity; MVPA, moderate-to-vigorous physical activity; CI, confidence interval

There were significant interactions of physical behaviours (*p* < 0.05, excepted for the associations of sleep onset with the physical activity chronotype, of duration of sleep window with MVPA, and of sleep efficiency with all physical behaviours) and daylight exposure (*p* < 0.05, excepted for sleep efficiency) with sex but not with season of accelerometer wear (all *p* > 0.05). Overall, associations of physical behaviours and daylight variables with sleep were stronger in women as compared to men (Fig. 3).

## Discussion

This study examining the day-to-day association of objectively assessed physical behaviours and daylight exposure with sleep among 3942 participants presents three key findings. First, increased proportion of sedentary behaviour during the day was associated with shorter sleep duration during the night while increased physical activity was associated with longer sleep duration. Second, those exposed longer to daylight > 1000 lx also had longer sleep duration. Third, associations of physical behaviours and daylight exposure with sleep duration were independent from each other.

### Comparison with previous studies

Our findings were similar to those of two studies that examined day-to-day association between objective measures of physical behaviours and sleep among older adults [17, 18]. One based in older women (mean age = 73.3 ± 1.7 years) suggested that a 10% increase in daily MVPA was associated with an increase of 0.2 to 0.4% of sleep duration [18]. Another study based on middle-aged and older adults (age range: 53 to 101 years) reported that 10% increase in MVPA was associated with an increase of 6 minutes in sleep duration [17]. These estimates are consistent with our results showing that spending 10% of the waking window more in MVPA was associated with 2 to 3 min longer sleep duration, according to adjustment level. In contrast, a study based on middle to old age adults (age range: 45 to 86 years) reported weekly averaged physical activity and sedentary behaviour to be associated with sleep efficiency and timing but not duration [23]. In another study, LIPA, but not MVPA, was associated with better sleep quality such as higher efficiency and lower fragmentation [21]. In the present study, both LIPA and MVPA were associated with sleep duration and timing with effect sizes in the same range, although the association with MVPA was attenuated once daylight exposure was considered.

Light is considered as a key determinant of the sleep-wake cycle [14, 26]. This association is suggested to depend on light exposition parameters such as light intensity [14], chronotype [14], or the wavelength [45]. Bright light has been shown to be more strongly associated with self-reported sleep positive outcomes than less intense light [14]. Also, exposition to bright light in the morning seems to be associated with sleep improvements [14] in contrast to evening exposition, which may delay the biological clock [46]. It has been reported that exposition to light during the 4 hours before bed time delays sleep onset [46]. However, the association between light and sleep is not clearly established among older adults due to the paucity of studies in this age group [14]; the existing evidence is based mainly on studies with small sample size [46], experimental design [45], or highly variable methodological approaches [14]. Our findings based on older adults confirm previous findings, showing both that the proportion of waking windows spent in light > 1000 lx was associated with longer sleep duration and that people exposed to light in the afternoon or evening fell asleep later than those more exposed in the morning.

Recent reviews of the literature suggest that the association between physical activity and sleep characteristics is similar in men and women [15, 47]. In the present study, the day-to-day associations of physical behaviours and daylight exposure with sleep outcomes were stronger in women. The pattern of physical behaviours [28]and sleep [29] tend to differ by sex and how this affects the physical behaviours-sleep association remained to be elucidated. It is reported that women are more likely to have seasonal affective disorder than men [48], suggesting they might be more sensitive to daylight exposure and its effects on sleep. Further studies are needed to investigate whether interventions including physical behaviours and daylight exposure might be more relevant to improve sleep among women than men.

The present study provides new findings. First, physical activity, sedentary behaviour, and daylight were independently associated with sleep characteristics. Part of these associations was attenuated when considering both physical activity and daylight exposure, highlighting the importance to consider both parameters when examining their associations with sleep. Two, we observed stronger associations for sleep quantity and timing than quality, where the larger effect size found for duration of sleep window than for time slept resulted in associations for sleep efficiency to be reversed as compared to those for sleep duration. Three, we explored the potential modifying role of sex and season on the associations of physical behaviours and daylight with sleep and found associations to be stronger among women than men but not to be affected by season of wear. Finally, despite the consistent associations of physical behaviours and daylight with sleep characteristics, the effect sizes were small and not clinically relevant – mean increases of 1 h38 (10% of mean waking windows) of MVPA and exposure to daylight > 1000 lx were associated with 2.2 and 1.5 minutes longer sleep duration – as compared to other risk factors such as smoking or obesity. This effect extended to 6 minutes among those with sleep efficiency< 80%. Individuals with sleep problems slept on average 5 h30 to 6 h28 (according to the different definitions of sleep problems) in our sample and the minimum recommended sleep duration is 7 hours. This suggests that interventions focussing only on physical behaviours and daylight exposure might not be enough to clinically improve sleep among older adults.

### Biological hypotheses underlying the association between physical behaviours, daylight and sleep

Hypotheses have been proposed to explain the association between physical activity and sleep. Exercise contributes to the regulation of body temperature, mood, and cardiac, autonomic, metabolic, and endocrine functions during sleep [49, 50]. Other explanations rely on the role of physical activity for the regulation of circadian rhythm, closely linked to sleep components [50]. The role of daylight for sleep is mainly documented in the context of circadian rhythm [51]. Outdoor natural light is suggested to influence wake/sleep cycle via retinal photoreceptors, the suprachiasmatic nuclei, and their role in release of sleep hormones such as melatonin [40]. In addition, exposition to non-natural light at night could lead to alteration of the wake/sleep cycle by shifting the peak of release of melatonin at later time and consequently delaying the circadian rhythm chronotype [45, 52].

### Strengths and limitations

This study has several strengths. Physical behaviours, daylight, and sleep were assessed objectively over a period of a week. Models were adjusted for a broad range of potential confounders, some of them not having been previously considered such as the seasonality although its role in the association investigated has been suggested [53]. Several methodological aspects also need to be highlighted. First, we used proportions of waking window rather than absolute durations in physical behaviours or light exposure to address their dependency with sleep time resulting from the finite nature of the 24-hour day duration [17, 23, 24]. Second, we assessed day-to-day association of physical behaviours and light with sleep instead of average data over several days. This approach allows to account for the temporality of the association given the suggested bidirectional association between physical behaviours and sleep [17, 18].

Findings of this study also need to be interpreted in the light of its limitations. Wrist accelerometers do not assess posture, not allowing differentiation between sitting and standing. This could lead to some misclassification between SB and less intense LIPA [54]. However, wrist accelerometers are reported to accurately classify physical behaviours based on metabolic intensity [55]. Despite the fact that the accelerometer assessed light from the wrist and not directly from the eyes, the literature reports a good reliability of the measurement of exposure to outdoor light at the set threshold, 1000 lx [31]. Second, our focus was on day-to-day association between physical behaviours and sleep. However, it is possible that chronic physical activity over the lifecourse is a stronger predictor of sleep in old age or that physical activity on 1 day impacts differently sleep on the same day and sleep on next days, given the delayed onset muscle soreness [56]. Future studies with a different study design and longer observational period are required to investigate these research questions. Finally, the Whitehall II study is an occupational cohort wherein participants are healthier than the general population, but it has been shown previously that the associations of risk factors and health were similar to those found in the general population [57].

### Meaning of the study and conclusion

This study based on a large sample of older adults suggests that physical activity and daylight exposure are independently associated with longer sleep duration, with associations being stronger among women than in men. The small effect sizes of the observed associations suggest that physical activity and daylight exposure might not be enough to improve sleep considerably. Instead, they may be added as one of many targets in a multidimensional sleep intervention strategy alongside others such as smoking cessation and weight loss management.

## Supplementary Information

**Additional file 1: Supplementary Figure 1**. Day-to-day association of physical behaviours and daylight exposure with sleep characteristics: additional adjustment for chronotype. **Supplementary Figure 2**. Day-to-day association of physical behaviours and daylight exposure with sleep characteristics: sensitivity analysis excluding participants using sleep medications and those with depression (*N* = 3154). **Supplementary Figure 3**. Independent day-to-day association of physical behaviours and daylight exposure with sleep characteristics among those with mild sleep problems (Definition 1, Jenkins sleep problem score ≥ 12, *N* = 529). **Supplementary Figure 4**. Independent day-to-day association of physical behaviours and daylight exposure with sleep characteristics among those with mild sleep problems (Definition 2, accelerometer-derived sleep efficiency< 80%, *N* = 367). **Supplementary Table 1**. Mean (standard deviation) of physical behaviours and daylight exposure by medians of person-level estimates of sleep characteristics (*N* = 3942). **Supplementary Table 2**. Day-to-day association of physical behaviours and daylight exposure with sleep characteristics. **Supplementary Table 3**. Association between covariates and sleep characteristics.

## Data Availability

The Whitehall II data cannot be shared publicly because of constraints dictated by the study’s ethics approval and institutional review board restrictions. The Whitehall II data are available for sharing within the scientific community. Researchers can apply for data access at https://www.ucl.ac.uk/epidemiology-health-care/research/epidemiology-and-public-health/research/whitehall-ii/data-sharing.

## Abbreviations

BMI: Body Mass Index
LIPA: Light-Intensity Physical Activity
MVPA: Moderate-to-Vigorous Physical Activity
SB: Sedentary Behaviour
