# Supplementary material for: Association of physical activity, sedentary behaviour, and daylight exposure with sleep in an ageing population: findings from the Whitehall accelerometer sub-study
Source: Int J Behav Nutr Phys Act. 2022 Dec 9;19:144. doi: 10.1186/s12966-022-01391-0 (PMC9733167; doi:10.1186/s12966-022-01391-0)
Supplement: Supplementary file 1 — Additional file 1: Supplementary Figure 1. Day-to-day association of physical behaviours and daylight exposure with sleep characteristics: additional adjustment for chronotype. Supplementary Figure 2. Day-to-day association of physical behaviours and daylight exposure with sleep characteristics: sensitivity analysis excluding participants using sleep medications and those with depression (N = 3154). Supplementary Figure 3. Independent day-to-day association of physical behaviours and daylight exposure with sleep characteristics among those with mild sleep problems (Definition 1, Jenkins sleep problem score ≥ 12, N = 529). Supplementary Figure 4. Independent day-to-day association of physical behaviours and daylight exposure with sleep characteristics among those with mild sleep problems (Definition 2, accelerometer-derived sleep efficiency< 80%, N = 367). Supplementary Table 1. Mean (standard deviation) of physical behaviours and daylight exposure by medians of person-level estimates of sleep characteristics (N = 3942). Supplementary Table 2. Day-to-day association of physical behaviours and daylight exposure with sleep characteristics. Supplementary Table 3. Association between covariates and sleep characteristics. [file 12966_2022_1391_MOESM1_ESM.docx]

**Supplementary Figure 1.** Day-to-day association of physical behaviours and daylight exposure with sleep characteristics: additional adjustment for chronotype


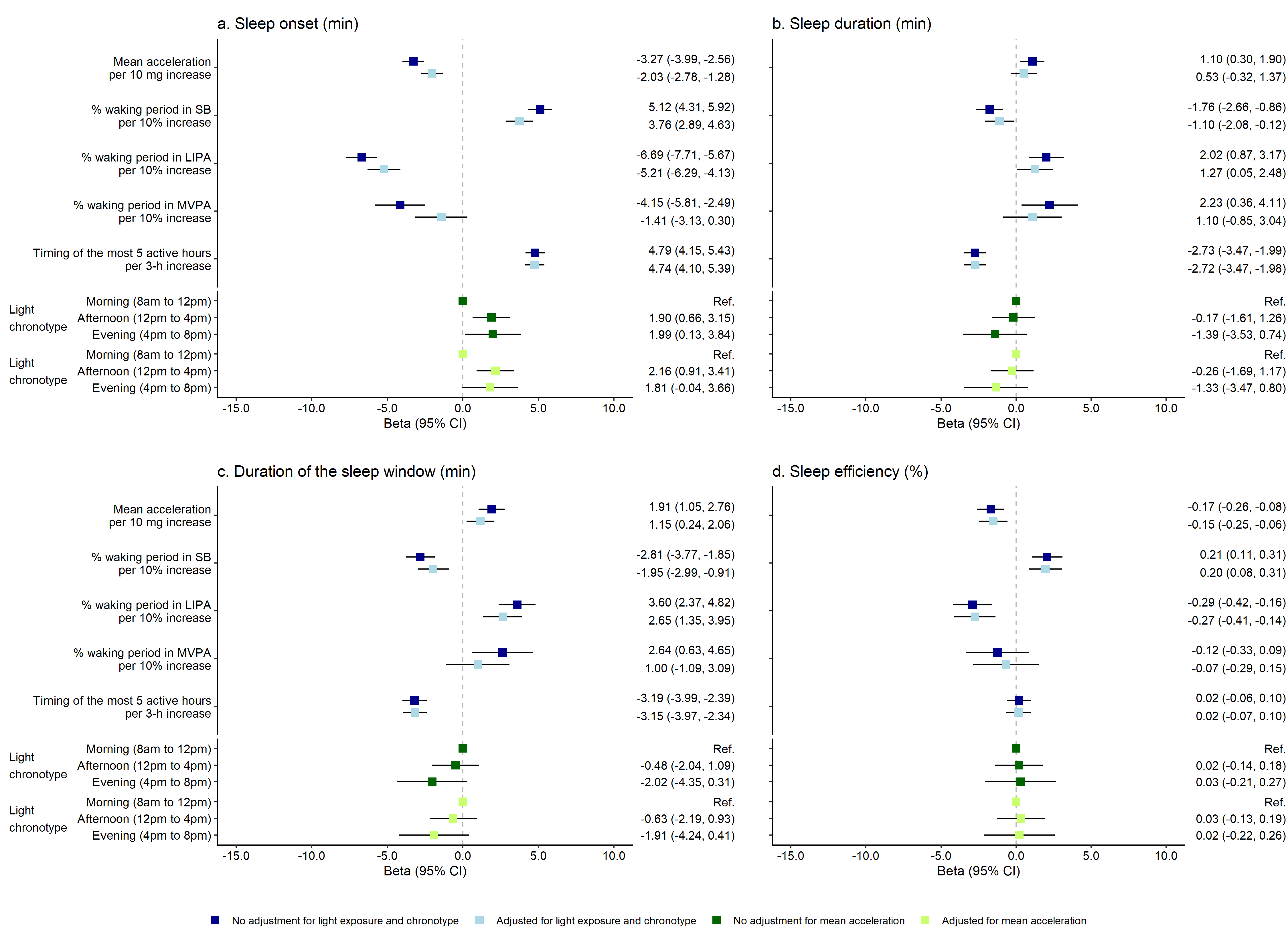


Models are adjusted for sociodemographic, behavioural, health-related factors, without or with additional adjustment for mean acceleration or light as indicated by the legend. Abbreviations: SB, sedentary behaviour; LIPA, light physical activity; MVPA, moderate-to-vigorous physical activity; CI, confidence interval.

**Supplementary Figure 2.** Day-to-day association of physical behaviours and daylight exposure with sleep characteristics: sensitivity analysis excluding participants using sleep medications and those with depression (N=3154)


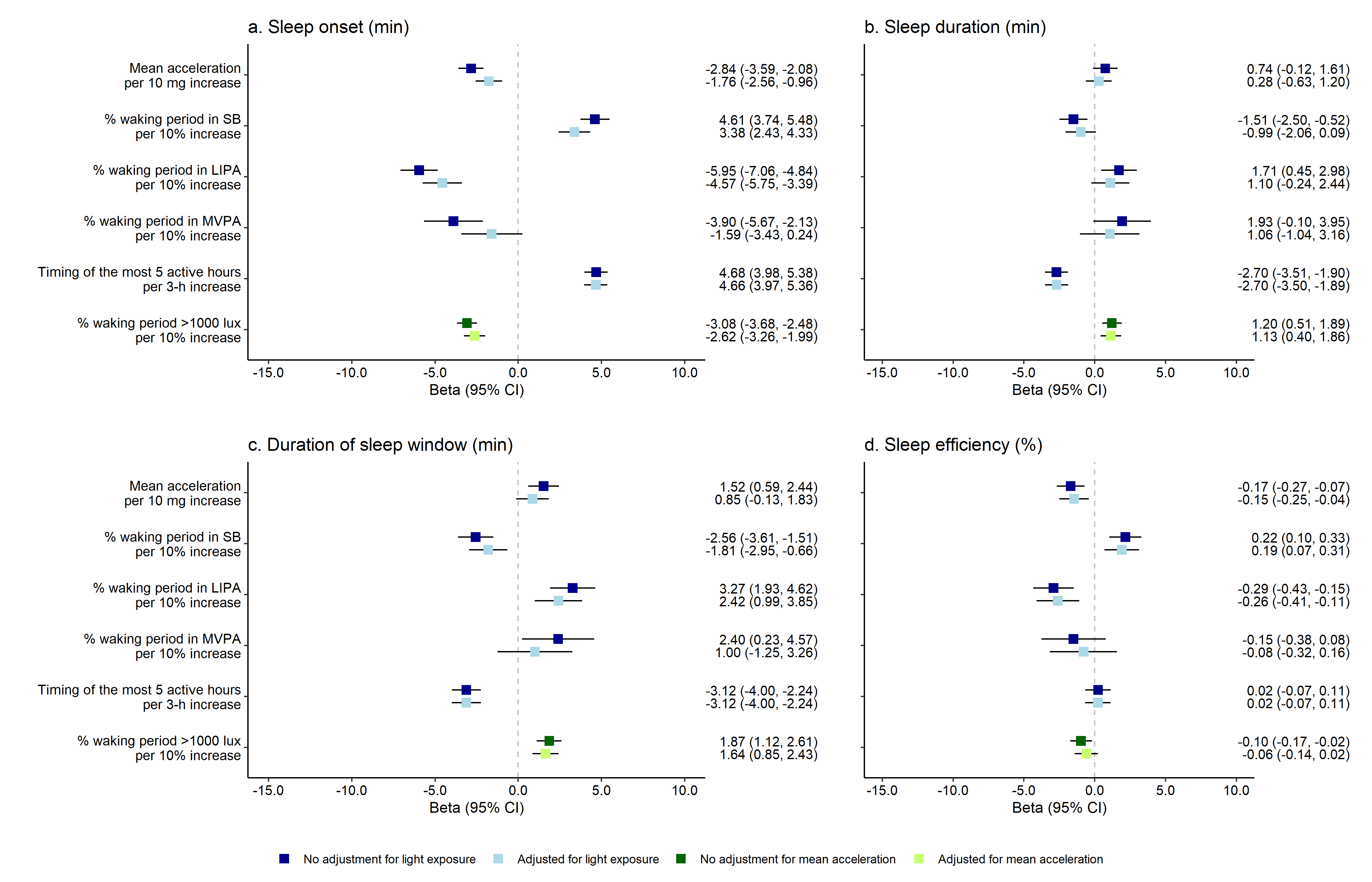


Models are adjusted for sociodemographic, behavioural, health-related factors, without or with additional adjustment for mean acceleration or light as indicated by the legend. Abbreviations: SB, sedentary behaviour; LIPA, light physical activity; MVPA, moderate-to-vigorous physical activity; CI, confidence interval.

**Supplementary Figure 3.** Independent day-to-day association of physical behaviours and daylight exposure with sleep characteristics among those with mild sleep problems (Definition 1, Jenkins sleep problem score≥12, N=529)


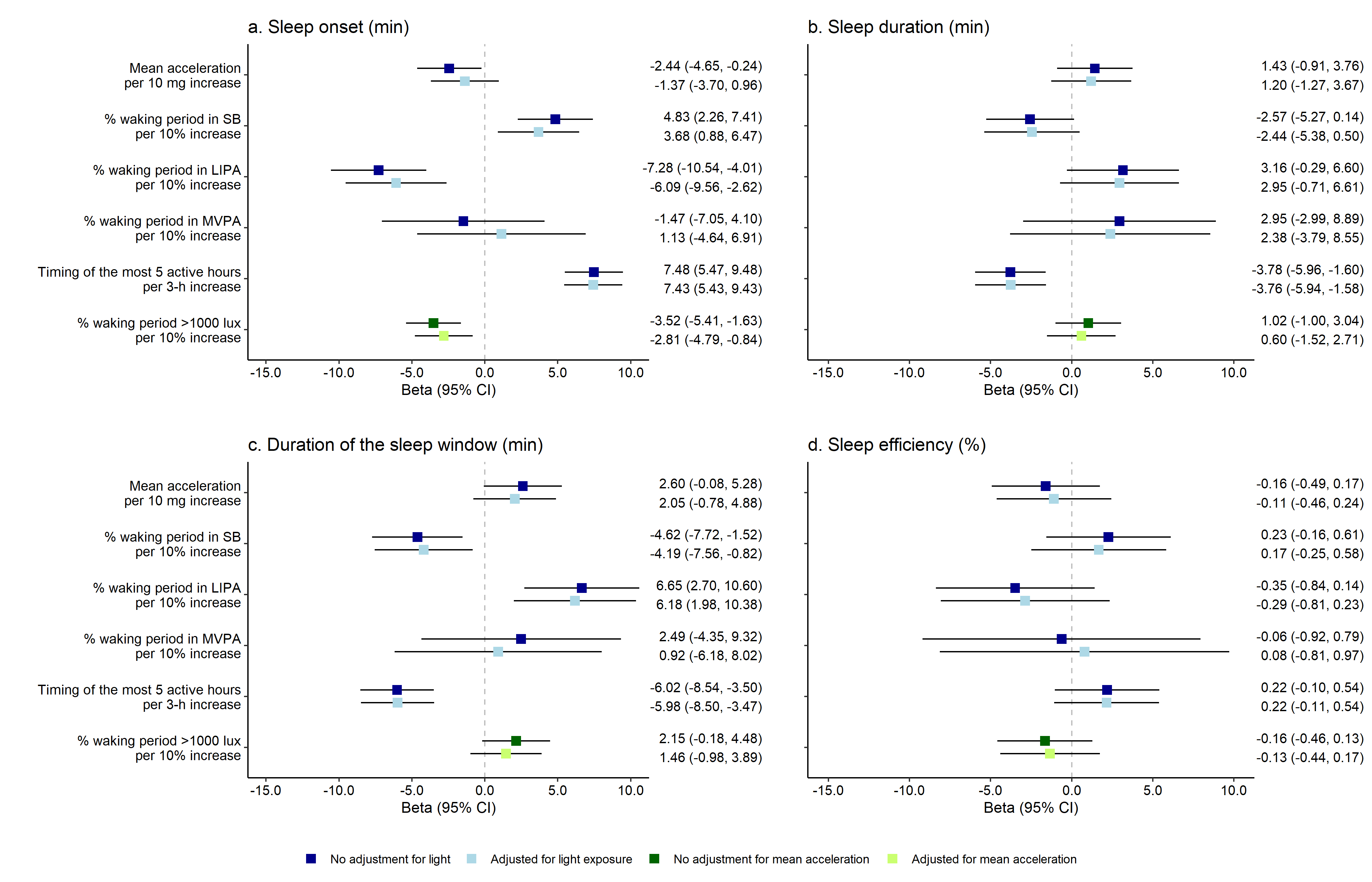


Models are adjusted for sociodemographic, behavioural, health-related factors, without or with additional adjustment for mean acceleration or light, as indicated in the legend.

**Supplementary Figure 4.** Independent day-to-day association of physical behaviours and daylight exposure with sleep characteristics among those with mild sleep problems (Definition 2, accelerometer-derived sleep efficiency<80%, N=367)


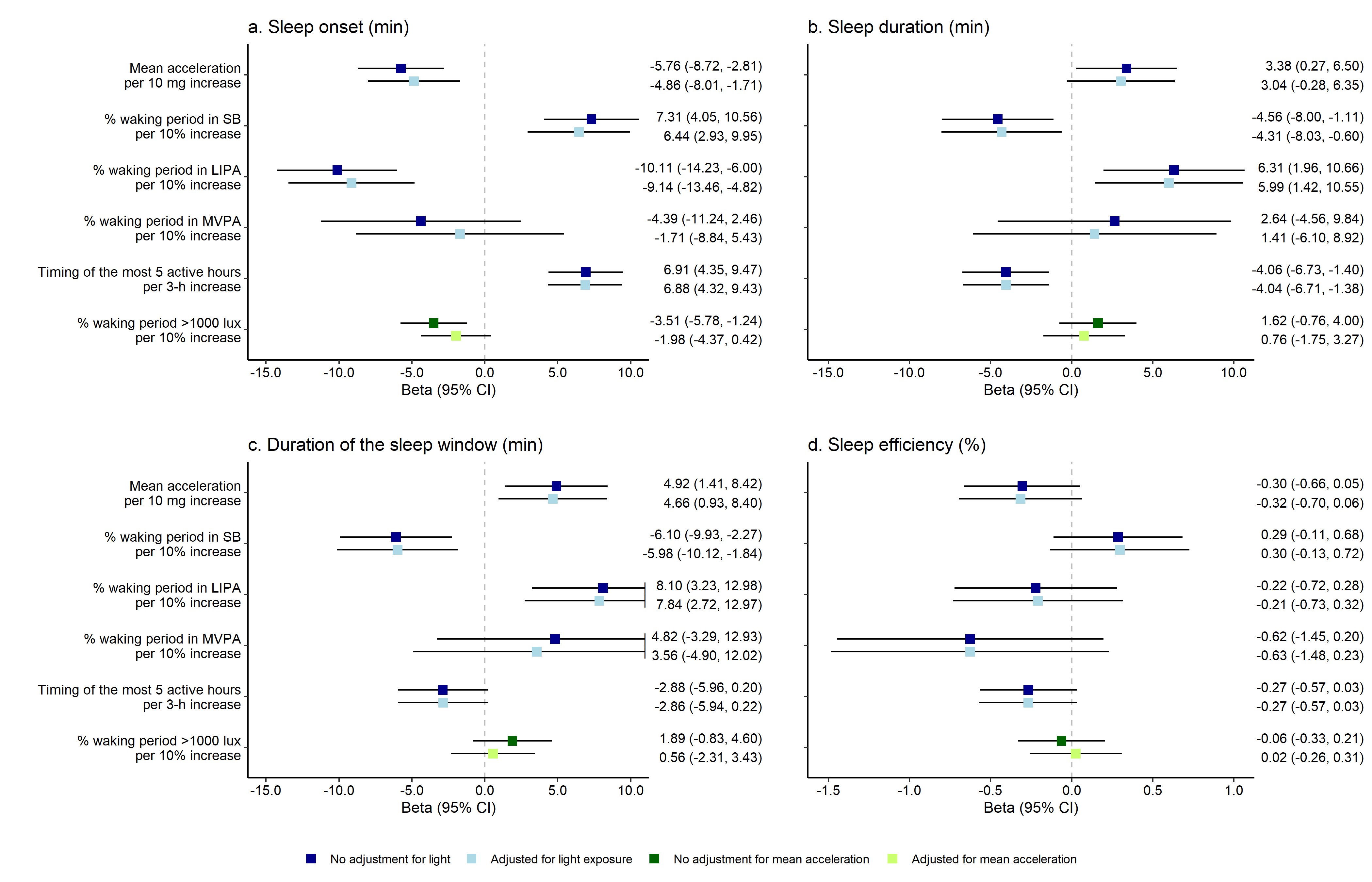


Models are adjusted for sociodemographic, behavioural, health-related factors, without or with additional adjustment for mean acceleration or light, as indicated in the legend.

**Supplementary Table 1.** Mean (standard deviation) of physical behaviours and daylight exposure by medians of person-level estimates of sleep characteristics (N=3942)

|  | **Sleep onset ^a^** | | |  | **Sleep duration ^a^** | | |  | **Duration of sleep window ^a^** | | |  | **Sleep efficiency ^a^** | | |
| --- | --- | --- | --- | --- | --- | --- | --- | --- | --- | --- | --- | --- | --- | --- | --- |
| **Characteristics** | <23:43 ^b^ | ≥23:43 ^b^ | *p* |  | <6h 39min ^b^ | ≥6h 39min ^b^ | *p* |  | <7h 38min ^b^ | ≥7h 38min ^b^ | *p* |  | <88% ^b^ | ≥88% ^b^ | *p* |
| Mean acceleration (m*g*) | 32.3 (9.9) | 31.5 (9.6) | 0.015 |  | 31.8 (9.7) | 32.0 (9.8) | 0.635 |  | *32.2 (9.4)* | *31.6 (10.0)* | *0.035* |  | 32.0 (10.1) | 31.8 (9.3) | 0.655 |
| % waking period in SB | 72.3 (9.5) | 73.4 (9.3) | <0.001 |  | 72.9 (9.4) | 72.8 (9.4) | 0.618 |  | 72.6 (9.3) | 73.1 (9.5) | 0.082 |  | 72.8 (9.6) | 72.9 (9.2) | 0.518 |
| % waking period in LIPA | 21.8 (6.9) | 21.0 (6.9) | <0.001 |  | 21.4 (7.0) | 21.4 (6.9) | 0.929 |  | 21.6 (6.9) | 21.3 (7.0) | 0.235 |  | 21.6 (7.1) | 21.3 (6.8) | 0.151 |
| % waking period in MVPA | 5.8 (4.0) | 5.6 (3.8) | 0.089 |  | 5.7 (3.9) | 5.8 (4.0) | 0.297 |  | *5.8 (3.8)* | *5.6 (4.0)* | *0.037* |  | 5.7 (4.0) | 5.8 (3.9) | 0.325 |
| Timing of the five most active hours (hours) | 9.8 (1.5) | 10.7 (1.6) | <0.001 |  | 10.2 (1.7) | 10.3 (1.5) | 0.178 |  | 10.2 (1.7) | 10.3 (1.5) | 0.294 |  | 10.3 (1.6) | 10.2 (1.6) | 0.309 |
| % waking window > 1000 lux | 15.1 (13.3) | 12.3 (11.5) | <0.001 |  | 13.1 (12.1) | 14.2 (12.9) | 0.005 |  | 13.5 (12.3) | 13.9 (12.7) | 0.246 |  | 13.7 (12.7) | 13.7 (12.4) | 0.984 |

Abbreviations: SB: sedentary behaviour; LIPA: light-intensity physical activity; MVPA: moderate-to-vigorous physical activity.

^a^ Data were averaged over the days of the observation period.

^b^ Median value in the study sample.

**Supplementary Table 2.** Day-to-day association of physical behaviours and daylight exposure with sleep characteristics

|  | **Sleep onset (min)** | |  | **Sleep duration (min)** | |  | **Duration of sleep window (min)** | |  | **Sleep efficiency (%)** | |
| --- | --- | --- | --- | --- | --- | --- | --- | --- | --- | --- | --- |
|  | Beta (95% CI)^a^ | p |  | Beta (95% CI)^a^ | p |  | Beta (95% CI)^a^ | p |  | Beta (95% CI)^a^ | p |
| **% waking window in SB** | | | | | | | | | | | |
| Tertile 3 | Ref |  |  | ref |  |  | ref |  |  | ref |  |
| Tertile 2 | -5.10 (-6.75, -3.46) | <0.001 |  | 1.70 (-0.18, 3.58) | 0.076 |  | 2.23 (0.20, 4.26) | 0.032 |  | -0.15 (-0.36, 0.06) | 0.155 |
| Tertile 1 | -11.39 (-13.36, -9.42) | <0.001 |  | 4.46 (2.24, 6.69) | <0.001 |  | 6.20 (3.82, 8.58) | <0.001 |  | -0.31 (-0.56, -0.06) | 0.016 |
| **% waking window in LIPA** | | | | | | | | | | | |
| Tertile 1 | ref |  |  | ref |  |  | ref |  |  | ref |  |
| Tertile 2 | -4.88 (-6.48, -3.28) | <0.001 |  | 1.61 (-0.22, 3.44) | 0.086 |  | 2.69 (0.71, 4.68) | 0.008 |  | -0.24 (-0.44, -0.04) | 0.021 |
| Tertile 3 | -11.09 (-12.99, -9.19) | <0.001 |  | 3.79 (1.64, 5.95) | <0.001 |  | 6.48 (4.17, 8.80) | <0.001 |  | -0.45 (-0.69, -0.21) | <0.001 |
| **% waking window in MVPA** | | | | | | | | | | | |
| Tertile 1 | ref |  |  | ref |  |  | ref |  |  | ref |  |
| Tertile 2 | -1.22 (-2.86, 0.43) | 0.147 |  | 1.14 (-0.74, 3.01) | 0.235 |  | 0.59 (-1.44, 2.62) | 0.569 |  | 0.03 (-0.18, 0.24) | 0.755 |
| Tertile 3 | -3.94 (-5.91, -1.98) | <0.001 |  | 2.06 (-0.16, 4.28) | 0.069 |  | 1.31 (-1.07, 3.70) | 0.281 |  | 0.09 (-0.16, 0.34) | 0.494 |
| **Mean acceleration** | | | | | | | | | | | |
| Tertile 1 | ref |  |  | ref |  |  | ref |  |  | ref |  |
| Tertile 2 | -5.48 (-7.12, -3.84) | <0.001 |  | 2.46 (0.59, 4.34) | 0.010 |  | 3.82 (1.79, 5.85) | <0.001 |  | -0.26 (-0.47, -0.05) | 0.014 |
| Tertile 3 | -9.95 (-11.90, -7.99) | <0.001 |  | 5.03 (2.82, 7.24) | <0.001 |  | 6.57 (4.20, 8.94) | <0.001 |  | -0.23 (-0.47, 0.02) | 0.075 |
| **Timing of the most 5 active hours** | | | | | | | | | | | |
| Tertile 1 | ref |  |  | ref |  |  | ref |  |  | ref |  |
| Tertile 2 | 4.89 (3.42, 6.37) | <0.001 |  | -1.01 (-2.70, 0.69) | 0.244 |  | -0.91 (-2.75, 0.93) | 0.334 |  | 0.05 (-0.14, 0.24) | 0.617 |
| Tertile 3 | 8.30 (6.80, 9.81) | <0.001 |  | -4.03 (-5.75, -2.30) | <0.001 |  | -4.41 (-6.29, -2.53) | <0.001 |  | 0.02 (-0.17, 0.22) | 0.804 |
| **% waking window with light exposure>1000 lux** | | | | | | | | | | | |
| Tertile 1 | ref |  |  | ref |  |  | ref |  |  | ref |  |
| Tertile 2 | -3.24 (-4.76, -1.72) | <0.001 |  | 0.79 (-0.95, 2.54) | 0.374 |  | 1.43 (-0.47, 3.33) | 0.141 |  | -0.04 (-0.23, 0.16) | 0.714 |
| Tertile 3 | -8.48 (-10.39, -6.57) | <0.001 |  | 3.84 (1.66, 6.02) | <0.001 |  | 5.03 (2.67, 7.39) | <0.001 |  | -0.05 (-0.29, 0.20) | 0.713 |

Abbreviations: SB, sedentary behaviour; LIPA, light physical activity; MVPA, moderate-to-vigorous physical activity; CI, confidence interval.

^a^Estimated using linear mixed model regressions.

Models are adjusted for sociodemographic factors, season of wear, and day type (week-end vs week day).

**Supplementary Table 3.** Association between covariates and sleep characteristics

|  | **Sleep onset (min)** | |  | **Sleep duration (min)** | |  | **Duration of sleep window (min)** | |  | **Sleep efficiency (%)** | |
| --- | --- | --- | --- | --- | --- | --- | --- | --- | --- | --- | --- |
|  | Beta (95% CI)^a^ | p |  | Beta (95% CI)^a^ | p |  | Beta (95% CI)^a^ | p |  | Beta (95% CI)^a^ | p |
| Age | -1.10 (-1.45, -0.76) | <0.001 |  | -0.06 (-0.40, 0.27) | 0.709 |  | 0.15 (-0.17, 0.47) | 0.350 |  | -0.04 (-0.09, 0.00) | 0.045 |
| Women | -1.44 (-6.07, 3.18) | 0.541 |  | 11.15 (6.66, 15.64) | <0.001 |  | 5.15 (0.82, 9.48) | 0.020 |  | 1.59 (1.02, 2.16) | <0.001 |
| Non-white | 1.59 (-5.55, 8.73) | 0.662 |  | -21.84 (-28.76, -14.92) | <0.001 |  | -1.81 (-8.48, 4.86) | 0.594 |  | -4.34 (-5.23, -3.46) | <0.001 |
| Level of education^b^ | 2.27 (0.56, 3.98) | 0.009 |  | -0.005 (-1.66, 1.65) | 0.995 |  | -0.91 (-2.51, 0.68) | 0.262 |  | 0.18 (-0.04, 0.39) | 0.104 |
| Married/cohabiting | 15.52 (11.16, 19.88) | <0.001 |  | -12.57 (-16.79, -8.34) | <0.001 |  | -11.89 (-15.96, -7.82) | <0.001 |  | -0.45 (-0.99, 0.09) | 0.100 |
| In employment | -8.48 (-13.22, -3.73) | <0.001 |  | -9.87 (-14.47, -5.27) | <0.001 |  | -11.56 (-15.99, -7.14) | <0.001 |  | 0.08 (-0.51, 0.67) | 0.795 |
| Weekend | 3.39 (2.24, 4.53) | <0.001 |  | 4.83 (3.50, 6.16) | <0.001 |  | 6.03 (4.58, 7.49) | <0.001 |  | -0.10 (-0.25, 0.05) | 0.182 |
| Summer or spring | 0.51 (-2.75, 3.78) | 0.757 |  | -6.35 (-9.70, -3.01) | <0.001 |  | -7.76 (-11.10, -4.43) | <0.001 |  | -0.02 (-0.42, 0.39) | 0.942 |
| Smoking status |  |  |  |  |  |  |  |  |  |  |  |
| Never smokers | 0.00 (ref) |  |  | 0.00 (ref) |  |  | 0.00 (ref) |  |  | 0.00 (ref) |  |
| Ex-smokers | 4.33 (0.60, 8.06) | 0.023 |  | -0.28 (-3.88, 3.33) | 0.881 |  | -1.17 (-4.64, 2.31) | 0.510 |  | 0.11 (-0.35, 0.58) | 0.629 |
| Current smokers | 25.95 (15.60, 36.30) | <0.001 |  | -8.00 (-18.03, 2.03) | 0.118 |  | -3.68 (-13.34, 5.98) | 0.456 |  | -1.25 (-2.53, 0.03) | 0.056 |
| Alcohol consumption |  |  |  |  |  |  |  |  |  |  |  |
| None in the past week | 0.00 (ref) |  |  | 0.00 (ref) |  |  | 0.00 (ref) |  |  | 0.00 (ref) |  |
| 1 - 14 units/week | 4.88 (0.04, 9.72) | 0.048 |  | -8.00 (-12.69, -3.32) | <0.001 |  | -6.00 (-10.51, -1.48) | 0.009 |  | -0.50 (-1.10, 0.10) | 0.100 |
| >14 units/week | 2.63 (-3.19, 8.45) | 0.375 |  | -6.89 (-12.53, -1.26) | 0.017 |  | -0.05 (-5.48, 5.38) | 0.985 |  | -1.44 (-2.16, -0.72) | <0.001 |
| Fruit & vegetable intake |  |  |  |  |  |  |  |  |  |  |  |
| Never or rarely | 0.00 (ref) |  |  | 0.00 (ref) |  |  | 0.00 (ref) |  |  | 0.00 (ref) |  |
| Once daily | -2.56 (-8.11, 2.99) | 0.366 |  | 1.94 (-3.43, 7.31) | 0.478 |  | 1.12 (-4.05, 6.29) | 0.671 |  | 0.15 (-0.54, 0.83) | 0.675 |
| Twice or more daily | -3.01 (-7.74, 1.72) | 0.212 |  | 3.37 (-1.21, 7.95) | 0.149 |  | -1.08 (-5.49, 3.33) | 0.632 |  | 0.89 (0.31, 1.48) | 0.003 |
| Having naps | 8.03 (4.21, 11.86) | <0.001 |  | -17.38 (-21.09, -13.68) | <0.001 |  | -16.24 (-19.80, -12.67) | <0.001 |  | -0.76 (-1.24, -0.29) | 0.002 |
| BMI |  |  |  |  |  |  |  |  |  |  |  |
| <25 kg/m² | 0.00 (ref) |  |  | 0.00 (ref) |  |  | 0.00 (ref) |  |  | 0.00 (ref) |  |
| 25-29.9 kg/m² | 5.49 (1.50, 9.48) | 0.007 |  | -7.17 (-11.04, -3.31) | <0.001 |  | -4.19 (-7.91, -0.47) | 0.027 |  | -0.82 (-1.31, -0.33) | 0.001 |
| ≥30 kg/m² | 5.69 (0.48, 10.89) | 0.032 |  | -14.09 (-19.14, -9.04) | <0.001 |  | -3.73 (-8.59, 1.14) | 0.134 |  | -2.39 (-3.03, -1.74) | <0.001 |
| Use of sleep medication | -0.69 (-5.94, 4.56) | 0.797 |  | 0.009 (-5.08, 5.09) | 0.997 |  | 4.92 (0.03, 9.82) | 0.049 |  | -0.99 (-1.64, -0.34) | 0.003 |
| Number of chronic conditions | -1.18 (-3.41, 1.05) | 0.300 |  | 1.86 (-0.30, 4.02) | 0.091 |  | 3.13 (1.05, 5.21) | 0.003 |  | -0.16 (-0.44, 0.11) | 0.245 |

Abbreviations: BMI: body mass index; CI, confidence interval.

^a^Estimated using linear mixed model regressions adjusted for all covariates, mean acceleration and % waking window with light exposure >1000 lux.

^b^Education level categorised as ≤primary school, lower secondary school, higher secondary school, university, or higher degree and treated as an ordinal variable.
